# Salt altered rhizosphere fungal community and induced soybean recruit specific species to ameliorate salt stress

**DOI:** 10.3389/fmicb.2023.1142780

**Published:** 2023-05-16

**Authors:** Ming Yuan, Di Zhang, Zhen Wang, Zhijia Zhu, Haoyue Sun, Wei Wang, Dezhi Han, Zhongcheng Qu, Bo Ma, Junqiang Wang, Lianxia Wang, Dongwei Han

**Affiliations:** ^1^Qiqihar Branch of Heilongjiang Academy of Agricultural Sciences, Qiqihar, China; ^2^Institute of Soil Fertilizer and Environmental Resources, Heilongjiang Academy of Agricultural Sciences, Harbin, China; ^3^Heihe Branch of Heilongjiang Academy of Agricultural Sciences, Heihe, China

**Keywords:** soybean, salt stress, fungal structure, network, recruitment

## Abstract

Different crop genotypes showed different adaptability to salt stress, which is partly attributable to the microorganisms in the rhizosphere. Yet, knowledge about how fungal communities of different genotypes in soybean respond to salt stress is limited. Here, qPCR and ITS sequencing were used to assess the response of rhizobial fungal communities of resistant and susceptible soybean to salt stress. Moreover, we isolated two fungal species recruited by resistant soybeans for validation. The assembly of fungal community structure might be strongly linked to alterations in fungal abundance and soil physicochemical properties. Salt stress derived structural differences in fungal communities of resistant and susceptible genotypes. The salt-resistant genotype appeared to recruit some fungal taxa to the rhizosphere to help mitigating salt stress. An increase of fungal taxa with predicted saprotrophic lifestyles might help promoting plant growth by increasing nutrient availability to the plants. Compared with the susceptible genotypes, the resistant genotypes had more stronger network structure of fungi. Lastly, we verified that recruited fungi, such as *Penicillium* and *Aspergillus,* can soybean adapt to salt stress. This study provided a promising approach for rhizospheric fungal community to enhance salt tolerance of soybean from the perspective of microbiology and ecology.

## Introduction

It is well known that soil salinization is a considerable problem in agricultural system, and that soil salinity can greatly reduce plant productivity and yield value (Khasanov et al., 2023). Due to an increase in global population and the ever-increasing demand for food quality, the issue of how to alleviate the pressure of soil salinity, improve plant resistance to salt stress and eventually increase crop yields is an urgent need to be addressed. Soybean [*Glycine max* (Linn.) Merr.], an important source of protein and oil in the world, is very sensitive to salt stress, which can severely restrict nutrient use and growth and development, ultimately reducing yields (Phang et al., 2008). In the last few years, traditional breeding techniques combined with beneficial microorganisms have been widely used to improve the salt resistance of soybeans (Pathan et al., 2007; Hanin et al., 2016).

Different soybean varieties have different root exudates, which determines the composition of the plant-specific root and microbial communities in rhizosphere area (Bulgarelli et al., 2013; Lian et al., 2019a). Under salt stress, the amount and type of root exudates secreted by different species are different (Lian et al., 2020). It has been demonstrated that salt-resistant soybeans have a much greater salicin, arbutin 6-phosphate, phosphoglycolate, and 1-methlseleno-N-acetyl-dgalactosamine than salt-susceptible soybeans in soils, which may increase the salt adaptation of soybeans (Lian et al., 2020).

Microorganisms have the benefit of promoting health and increasing productivity in plants (Mendes et al., 2013; Li et al., 2014a,b). Different types and amounts of metabolites from plants or microorganisms could alter the diversity and structure of rhizosphere microbes, which could assist the host to become more resistant to stress (Wu et al., 2006; Qin et al., 2016; Hu et al., 2018; Lian et al., 2020). It is well known that plant growth promoting rhizobacteria (PGPB) have certain functions that can promote plant growth (Bhatt et al., 2022). For example, a variety of metabolites produced by *Pseudomonas* can lead to salt stress-relieving, including exopolysaccharides, ACC deaminase and hormones (indoleacetic acid and gibberellins; Etesami and Glick, 2020; Li et al., 2021). However, studies in recent years have largely emphasized on bacteria, neglecting fungal species, with the improvement in nutrient cycling and the resistance to environment, which can also assist plants to mitigate damage caused by abiotic stresses (Kawai et al., 2000; Peltoniemi et al., 2012). *Penicillium* and *Aspergillus*, which were reported to increase nitrogen and phosphorus to plant roots, stimulate the growth of host plants by increasing the accumulation of nutrient under unfavorable conditions (Kiers et al., 2011), and thus might help plant alleviate the biotic and abiotic stresses. Thus, to understand how salt-resistant soybean better adapt to salt stress, it is necessary to investigate how rhizosphere microbes of salt-resistant soybean genotypes respond to salt stress.

In this study, we selected the resistant soybean (Qinong7) or susceptible soybean (Hefeng50), growing at soils under salt and non-salt stress. Then, we analyzed the fungal community structure in rhizosphere through ITS high-throughput sequencing. Moreover, the fungal community structure was investigated in relation to its physicochemical properties. We hypothesized that (1) Salt-R genotype possesses higher fungal diversity compared to Salt-S genotype, and (2) Salt-R genotype will enrich particular Salt-R fungal taxa to the rhizosphere that help mitigating salt stress.

## Materials and methods

### Pot experiment and rhizosphere soil collection

Two different soybean (*Glycine max L.*) genotypes were shown to be resistant (Qinong7) or susceptible (Hefeng50) to salt stress. Soil collection was conducted in an agricultural field in Qiqihar (110°25′N, 21°32′E), Heilongjiang Province, China in June 2022. We conducted a pot experiment with six replicates in a greenhouse at Heilongjiang Academy of Agricultural Sciences, Qiqihar Branch, Qiqihar, China, in a completely randomized block design. A 4 mm mesh was used to sieve the soil. Eight seeds were sown in each pot and then two better seedlings were kept after the ninth day of sowing. The temperature range of the greenhouse was 16–20°C night-time temperature and 25–30°C daytime temperature. Each treatment was watered with 150 mM NaCl solution, with equal amounts of pure water as a control. Soil moisture content was 85% of field capacity.

Soil samples were collected with a shaking of the root at the flowering stage. Each replicate for each treatment, a microcentrifuge tube containing 8 g of soil was placed at −80°C for DNA extraction after shaking for 3 min. Soil physicochemical property analysis were performed on the remaining soil that stored at 4°C.

### Soil properties admeasurement

Soil pH was measured using a pH metre. A VarioEL III elemental analyzer (Germany) was used to measure TN and TC contents in soil (Jones and Willett, 2006). Inductively coupled plasma atomic emission spectrometry (ICPS-7500, Shimadzu, Japan) was used to determine soil TK. A continuous flow analysis system (SKALAR SAN++, The Netherlands) was used to measure NH_4_^+^ -N and NO_3_^−^ -N, TP and Olsen-P.

### Molecular genetic analyses

Following the manufacturer’s instructions, DNA was extracted using a E.Z.N.A. DNA Kit for Soil (Omega, United States). qPCR was conducted through the ITS1F (5’-CTTGGTCATTTAGAGGAAGTAA-3′) and ITS2R (5’-GCTGCGTTCTTCATCGATGC-3′) primers to measure the fungal abundance (Jobst et al., 1998). PCR amplification system were as follows, with 15 μL of 2 × KAPA HiFi Mix, the forward and reverse primers (0.2 μM), and 0.5 ng of template DNA in a volume of 30 μL. PCR reaction cycling conditions were followed by 3 min at 95°C for one cycle, 30 s at 95°C, 30 s at 55°C, 15 s at 72°C for twenty-five cycles, and then 5 min at 72°C for thermal extension.

For next-generation sequencing, the hypervariable ITS region of fungal was amplified by ITS1F/ITS2R primers. Using an Illumina MiSeq platform, standard protocols were followed to paired-end sequence the pooled-purified in equimolar amounts of amplicons. PCR products were used to create sequencing libraries and then paired-end sequences were carried out on the Illumina MiSeq platform. Raw sequences were uploaded in the NCBI with accession number of PRJNA918498.

### Bioinformatic processing

Raw FASTQ files obtained by sequencing were subsequently used for processing in the QIIME Pipeline (version 1.19.1; Pauvert et al., 2019). To be brief, each sample was allocated to obtain a certain number of sequences reads, which were then quality filtered and chimeras were removed by UCHIME (Edgar et al., 2011). Based on the best match for the sequences in the RDP database, sequences were assigned phylogenetically by the RDP classifier (Wang et al., 2007). Amplicon sequence variants (ASVs) were classified using CD-HIT with 97% of sequence similarity (Li and Godzik, 2006). α-diversity (Chao1 richness and Shannon diversity) was done using QIIME.

Principal coordinate analysis (PCoA, Bray-Curtis’s dissimilarities), nonparametric multivariate analysis (PERMANOVA), were performed using the *aMDS* and *adonis* functions of the “vegan” package in R (version 4.0.2), respectively. Canonical correspondence analysis (CCA) and Mantel test were performed using *cca* and *mantel* functions of the “vegan” package in R, respectively (Anderson, 2001; Tierney, 2012; Oksanen et al., 2015). Fungal phyla relative abundance was indicated by the “circlize” package (Gu et al., 2014). Differential abundance analysis of 438 ASVs was carried out using generalized linear models and likelihood ratio tests to identify significant ASVs that caused the structural segregation of fungal communities across genotypes. ASVs that were co-enriched and unique across different salt treatments and genotypes were identified using Venn analysis (Yin et al., 2022). Statistical analyses were done by SPSS with Duncan tests at 95% confidence level (*p* < 0.05) (version 24.0, IBM, United States).

To understand the relationships in the fungal communities for each ASV, co-occurrence network analysis was performed in this study. ASVs with relative abundance exceeding 0.05% were to be selected for calculating the Spearman’s rank correlation coefficient between them. The standard for the determination of statistically significant correlation between ASVs was Spearman’s correlation coefficient more than 0.8 and *p* < 0.05 (Shi et al., 2020). Those nodes in the network were assigned at the phylum level of the fungus, and correlations were indicated by different colored edges, i.e., red was positive and green was negative. A series of indices were employed to evaluate the stability and complexity of the network, including graph density, the average weighted degree (avgK), the number of positive correlations, and modularity (M). The ASVs that with high betweenness centrality and high degree were consider as keystone species (Shi et al., 2020). After the statistical analyses completed, the Gephi was used for visualizing (Parente et al., 2016).

### Isolation of fungal species and their effect on the soybean

Microorganisms enriched in soybean grown under salt stress were considered to be salt-resistant. Samples of the rhizosphere were obtained by resuspension with 1 x phosphate buffer saline (PBS) (2.0 g of sample per 10 mL of PBS). The rhizosphere soil suspension was diluted to 10^−6^ and 100 μL dilutions and was plated to the fungal medium. 0.1 M NaCl was then added to the homogenate as an inoculum for microbial enrichment cultures via R2A liquid medium (at 30°C, 150 rpm on a rotary shaker for 48 h). After incubation for no more than 2 weeks, single colonies were selected from plates with no more than 20 colony forming units and subjected to ITS gene analysis. Then the single colonies were inoculated into susceptible soybeans at an inoculate level of 2 × 10^5^ per plant and soybean growth was observed 30 days after sowing.

## Results

### Biomass in soybean, fungal abundance, and diversity

**Figure 1:**
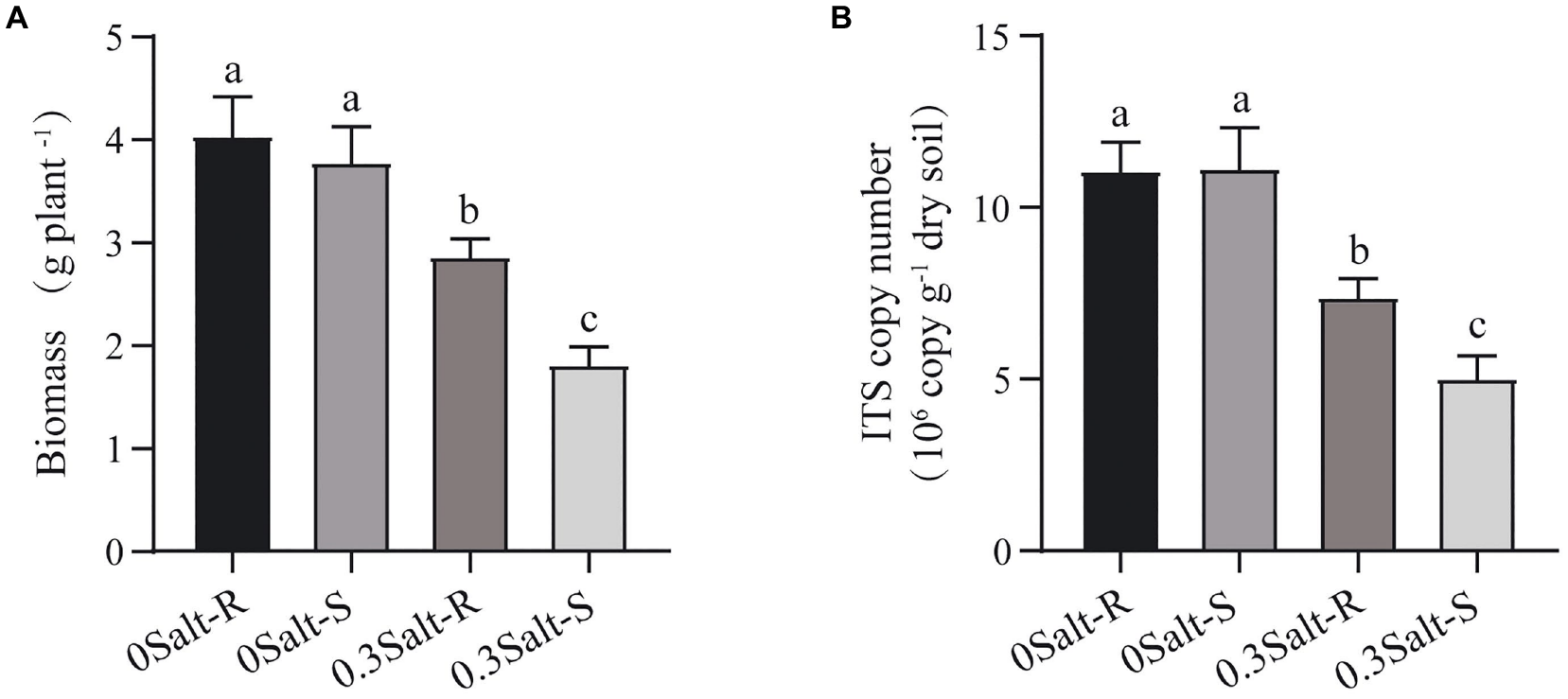
Effects of salt stress on soybean biomass **(A)**, and rhizospheric fungal ITS copy number **(B)** Salt-S and Salt-R were significant differed by one way ANOVA using Student’s t-tests (*p* < 0.05). The standard error of the biological repetition mean is shown through error bars (*n* = 6). Salt-R: salt-resistant soybean genotype; Salt-S: salt susceptible soybean genotype.

**Figure 2:**
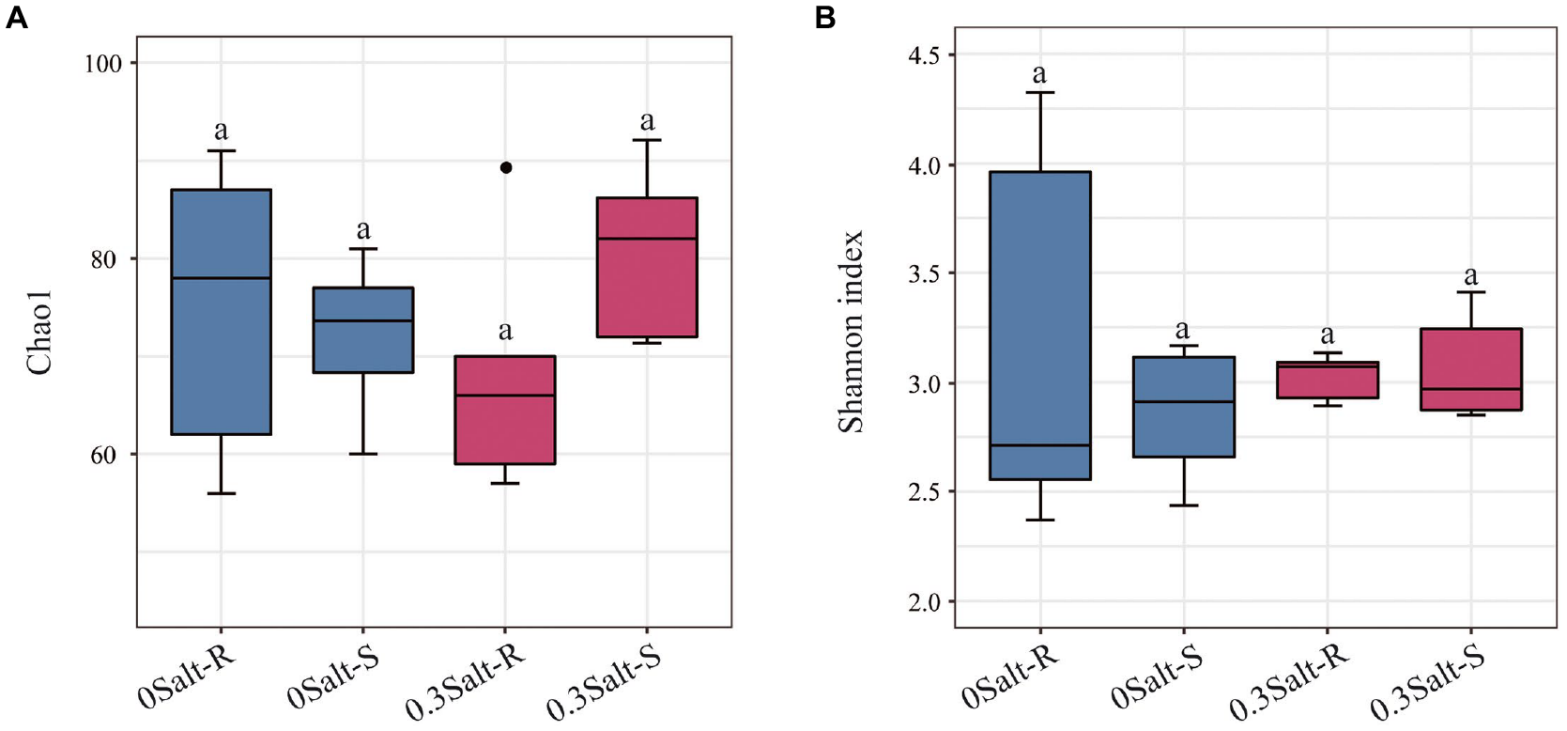
Effects of salt stress on Chao1 value **(A)** and Shannon index **(B)** of soybean rhizosphere. Salt-S and Salt-R were significant differed by one way ANOVA using Student’s t-tests (*p* < 0.05). The standard error of the biological repetition mean is shown through error bars. (*n* = 6). Salt-R: salt-resistant soybean genotype; Salt-S: salt susceptible soybean genotype.

Salt stress limited the increasement of soybean biomass in both genotypes and was more suppressive to Salt-S biomass than Salt-R (Figure 1A). In terms of fungal abundance, varying from 5.03 to 11.14 × 10^7^ copies g/dry soil, salt stress showed a significant decrease in the abundance of both genotypes and a higher abundance in Salt-R (Figure 1B, *p* < 0.05). Interestingly, salt stress exhibited no significantly effect on fungal Shannon diversity and Chao1 richness of Salt-R and Salt-S genotypes (Figure 2, *p* > 0.05).

### Fungal community structure in the rhizosphere

**Figure 3:**
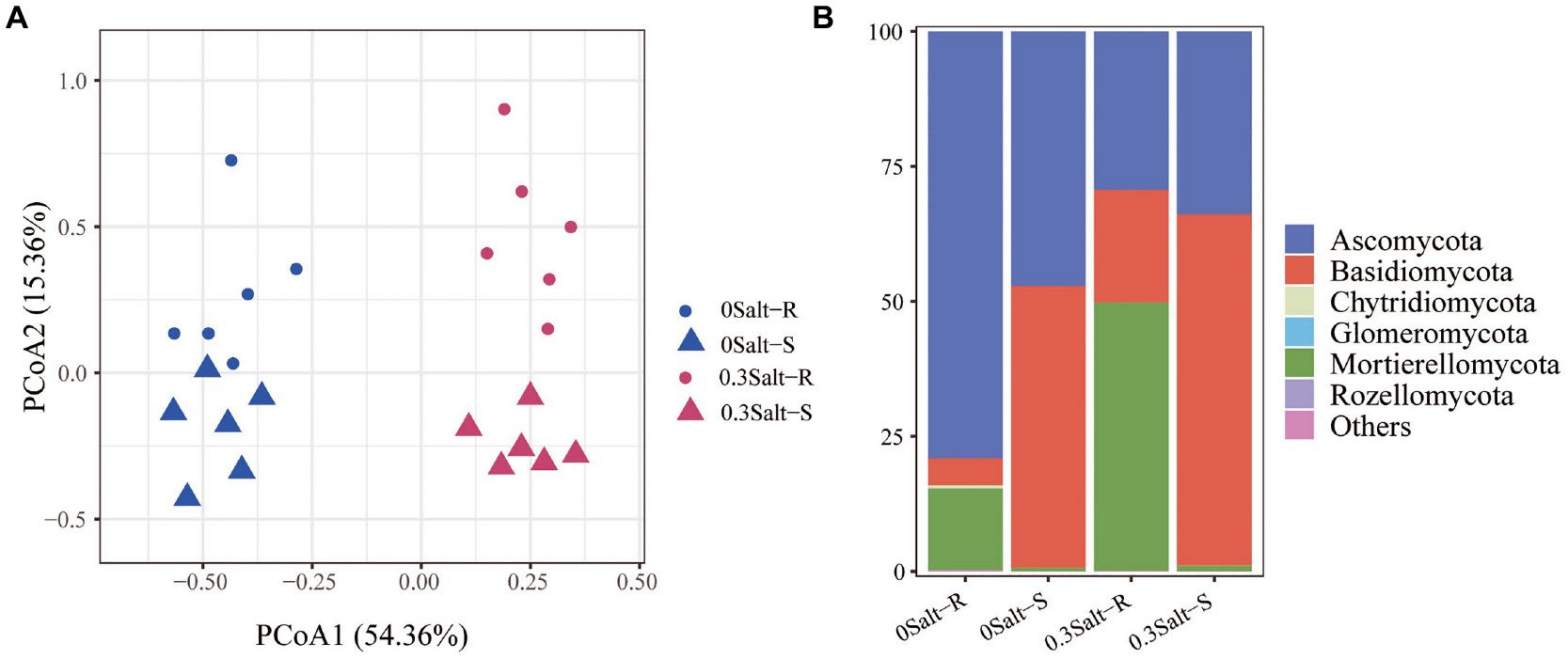
Principal co-ordinates analysis (PCoA) reveals the significant differences in the rhizospheric fungal community structures with and without salt stress **(A)**. The PERMANOVA F-ratios and *p*-values (n = 6) for the factor genotype are provided in the corner of the plots. Bar chart for calculating relative abundance of soybean rhizospheric fungal community on dominant phyla **(B)**. Salt-R: salt-resistant soybean genotype; Salt-S: salt susceptible soybean genotype.

There were 985,254 high qualities filtered fungal ITS1 sequences with a read number range of 52,531 ~ 96,735. Clustering yielded a total of 438 fungal ASVs. The fungal community structure was significantly dissimilar at the four treatment levels, but had a stronger tendency to detach under salt stress conditions rather than genotypes (Figure 3A). The fungal community members were assigned into six dominant phyla (Figure 3B). Ascomycota and Basidiomycota, followed by Chytridiomycota, Glomeromycota, Mortierellomycota and Rozellomycota were the main phyla across the treatments. In detail, for these six fungal phyla, relative abundance of Ascomycota was decreased with the salt stress, while the relative abundance of Mortierellomycota and Basidiomycota were increased in both Salt-R and Salt-S. Moreover, the relative abundance of Mortierellomycota was markedly higher in Salt-R than in Salt-S under salt stress.

**Figure 4:**
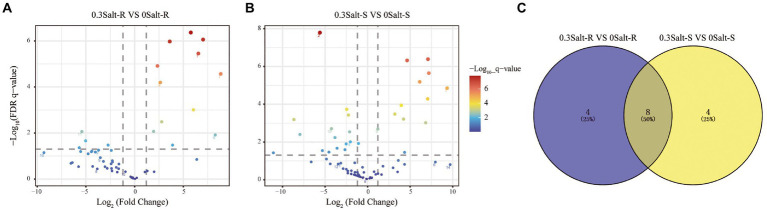
Enrichment and depletion of ASVs of Salt-R **(A)** and Salt-S **(B)**. Each point represents an independent ASV. Venn analysis was used to count the number of shared and unique enriched ASVs in the two comparison groups mentioned above **(C)**. Salt-R: salt-resistant soybean genotype; Salt-S: salt susceptible soybean genotype.

Using differential abundance analysis, each 12 ASVs were significantly enriched in Salt-R and Salt-S genotypes under salt stress, when compared to the control (Figure 4). Among them, four ASVs that ASV1 (*Saitozyma*), ASV6 (*Idriella*), ASV29 (*Talaromyces*) and ASV80 (*Cladosporium*) were specially enriched to Salt-R genotype soybean, while four ASVs that ASV44 (*Saitozyma*), ASV63 (*Cladosporium*), ASV75 (*Candida*) and ASV96 (*Gliomastix*) were specially enriched to Salt-S genotype soybean (Supplementary Tables S1, S2). Furthermore, eight ASVs that ASV7 (*Talaromyces*), ASV9 (*Saitozyma*), ASV17 (*Wallemia*), ASV19 (*Aspergillus*), ASV21 (*Wallemia*), ASV24 (*Saitozyma*), ASV32 (*Candida*) and ASV38 (*Papiliotrema*) were significantly enriched at both Salt-R and Salt-S genotypes (Supplementary Table S3).

**Figure 5:**
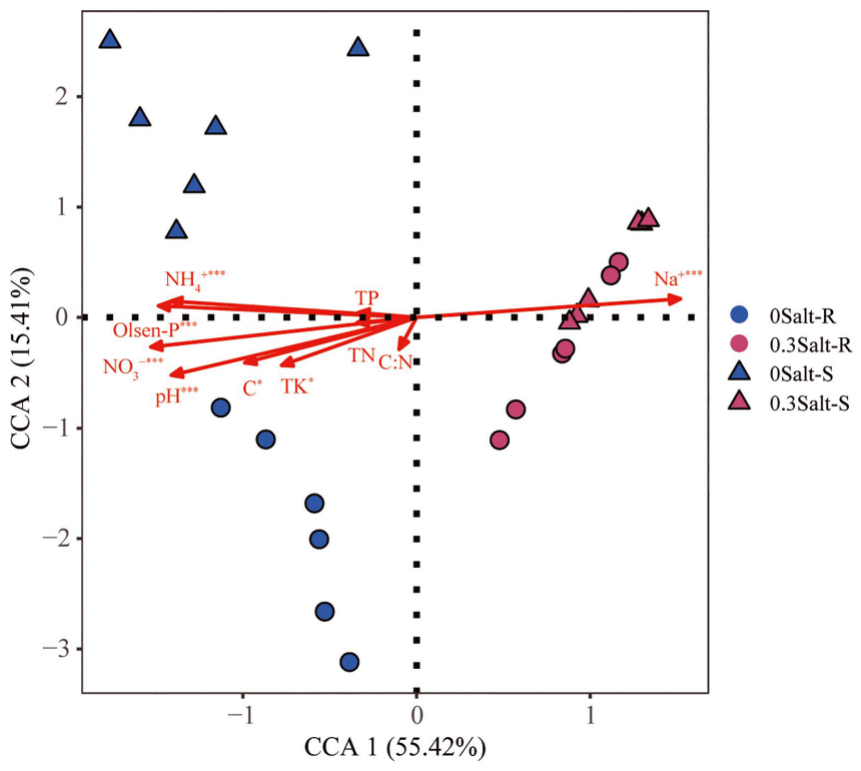
Canonical correspondence analysis (CCA) showed the relationship between rhizospheric fungal community structure and soil physicochemical factors.

The correlation linkages with soil physicochemical properties and fungal community structure were evaluated using CCA and Mantel test (Figure 5). The results indicated that the fungal communities of Salt-R and Salt-S genotypes showed significant correlations with certain soil physicochemical properties, including Na^+^, Olsen-P, pH, NH_4_^+^ and NO_3_^−^.

### FUNGuild analysis of rhizosphere soil fungi

All filtered ASVs were categorized into 10 guilds by FUNGuild (Table 1). With the exception of “Ectomycorrhizal,” “Fungal Parasite,” and “Animal Pathogen,” the remaining guilds differed significantly in terms of salt stress and genotypes. “Wood Saprotrophs” were the largest guild with 251 ASVs affiliated to Ascomycota and Basidiomycota, accounting for 32.6–51.8% in different genotypes. “Wood Saprotroph,” “Plant Pathogen,” “Endophyte” and “Soil Saprotroph” were significantly increased in the salt R genotype under the salt stress.

**Table 1:** Functional potentials of rhizospheric fungal community based on different salt stress and genotypes.

	0Salt-R	0Salt-S	0.3Salt-R	0.3Salt-S	*p* value
Wood Saprotroph	32.550 ± 4.795 c	36.687 ± 7.506 bc	51.808 ± 10.079 a	45.132 ± 13.642 ab	0.0107^*^
Undefined Saprotroph	33.873 ± 7.145 a	38.698 ± 10.415 a	17.040 ± 7.846 b	28.675 ± 7.037 a	0.0013^**^
Plant Pathogen	6.841 ± 4.219 b	4.289 ± 1.536 b	15.189 ± 10.558 a	6.4263 ± 3.828 b	0.0268^*^
Endophyte	1.418 ± 1.141 b	0.914 ± 0.312 b	8.679 ± 5.411 a	7.621 ± 5.495 a	0.003^**^
Ectomycorrhizal	0.244 ± 0.191 a	0.311 ± 0.218 a	0.371 ± 0.323 a	0.721 ± 0.645 a	0.1824
Fungal Parasite	0.379 ± 0.230 a	0.542 ± 0.188 a	0.952 ± 0.761 a	1.067 ± 0.850 a	0.1708
Soil Saprotroph	0.382 ± 0.637 b	2.279 ± 2.813 ab	3.591 ± 1.753 a	4.341 ± 1.519 a	0.0077^**^
Animal Pathogen	0.371 ± 0.287 ab	0.180 ± 0.081 ab	0.091 ± 0.0590 b	0.437 ± 0.414 a	0.1006
Arbuscular Mycorrhizal	0.007 ± 0.009 b	0.034 ± 0.021 a	0.003 ± 0.004 b	0.013 ± 0.018 b	0.0094^**^
Dung Saprotroph	0.128 ± 0.141 ab	0.213 ± 0.146 a	0.023 ± 0.017 b	0.027 ± 0.027 b	0.0125^*^
Unknown	23.800 ± 8.307 a	15.847 ± 3.914 b	2.248 ± 1.260 c	5.536 ± 2.435 c	0.0001^***^

^*^p < 0.05; ^**^p < 0.01; ^***^p < 0.001.

### Effect of salt on the fungal networks

**Figure 6:**
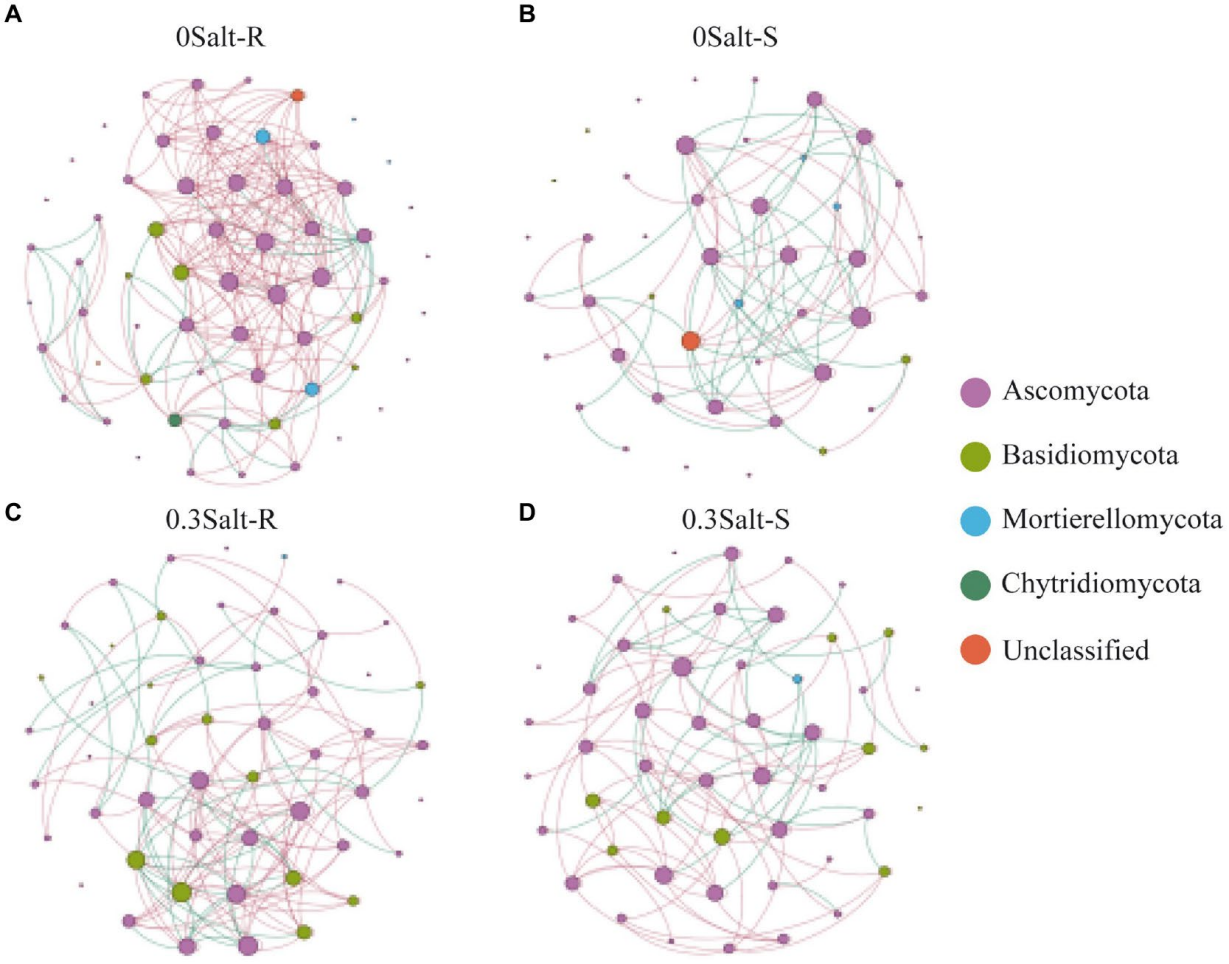
Co-occurrence network analysis of rhizospheric fungal communities. 0 Salt-R **(A)**, 0 Salt-S **(B)**, 0.3 Salt-R **(C)**, and 0.3 Salt-S **(D)**. OTUs are indicated by nodes, coloring-coded in phyla, and degree is indicated by node size. Lines shown in red indicate positive correlation (*r* > 0.8), lines shown in green indicate negative correlation (*r* < −0.8), and *p* < 0.05. Salt-R: salt-resistant soybean genotype; Salt-S: salt susceptible soybean genotype.

The co-occurrence network analysis of fungi in both genotypes showed that the network structure was significantly different by salt stress conditions (Figure 6; Table 2). Specifically, the number of nodes and positive correlations and average weighted degree (avgK) decreased in Salt-T genotype under salt stress, while the opposite trend was observed in Salt-S genotype. Interestingly, modularity (M) and average path length (APL) increased in Salt-T genotype and decreased in Salt-S genotype. Taken together, the salt-S genotype had a more intricate and stable network structure with respect to the salt-R genotype under salt stress. Degree of node, closeness centrality and betweenness centrality were the main indicators for identifying key ASVs (Table 3). For example, ASV45 (*Talaromyces*) and ASV8 (*Aspergillus*) were determined as keystones for the Salt-R genotype when exposed the salt stress.

**Table 2:** Network characteristics of rhizospheric microbial networks among different treatments.

Network characteristics	0Salt-R	0Salt-S	0.3Salt-R	0.3Salt-S
Number of nodes	61	44	51	51
Number of edges	276	119	170	153
Number of positive correlations	242	82	130	118
Number of negative correlations	34	37	40	35
Average path length	2.68	3.34	3.19	3.26
Graph density	0.15	0.13	0.13	0.12
Network Diameter	7	7	7	6
Average clustering coefficient	0.59	0.66	0.63	0.58
Average weighted degree	10.57	2.04	9.34	4.24
Connecting components	15	12	9	6
Modularity	0.40	14.85	1.29	1.46

**Table 3:** Keystones of the networks of different treatments.

Genotype	ASV	Degree	Closness centrality	Betweeness centrality	Phylum	Class	Order	Family	Genus	Species
0Salt-R	ASV12	19	0.505	74.348	Ascomycota	Sordariomycetes	Sordariales	Chaetomiaceae	*Humicola*	*Humicola_olivacea*
	ASV26	19	0.517	70.091	Ascomycota	Eurotiomycetes	Eurotiales	Trichocomaceae	*Talaromyces*	*Talaromyces_neofusisporus*
0Salt-S	ASV20	12	0.444	70.288	Ascomycota	Saccharomycetes	Saccharomycetales	Phaffomycetaceae	*Barnettozyma*	*Barnettozyma_californica*
	ASV23	11	0.475	103.343	Ascomycota	Eurotiomycetes	Eurotiales	Aspergillaceae	*Aspergillus*	*Aspergillus_pseudodeflectus*
0.3Salt-R	ASV45	14	0.423	74.713	Ascomycota	Eurotiomycetes	Eurotiales	Trichocomaceae	*Talaromyces*	*Talaromyces_helicus*
	ASV8	13	0.410	115.182	Ascomycota	Eurotiomycetes	Eurotiales	Aspergillaceae	*Aspergillus*	*Aspergillus_pseudodeflectus*
0.3Salt-S	ASV114	11	0.366	116.255	Ascomycota	Eurotiomycetes	Eurotiales	Aspergillaceae	*Aspergillus*	*Aspergillus_sepultus*
	ASV23	8	0.353	155.681	Ascomycota	Eurotiomycetes	Eurotiales	Aspergillaceae	*Aspergillus*	*Aspergillus_pseudodeflectus*

### Effect of *Talaromyces* and *Cladosporium* on the soybean under salt stress

More than 100 fungal clones were isolated and 15 strains were identified from the Salt-tolerant rhizosphere soil. Further comparing these 15 strains with the sequencing results, we found two strains, i.e., *Talaromyces* and *Cladosporium,* whose relative abundance was increased in the resistant genotpye. Pouring the fungal solution on the roots of soybean significantly increased the shoot and root biomass of soybean. In general, *Talaromyces* increased the shoot and root biomass of Salt-S soybeans by 21.9 and 19.7%, respectively, and *Cladosporium* increased the shoot and root biomass of Salt-S soybeans by 26.9 and 29.5%, respectively (Table 4).

**Table 4:** Effect of *Talaromyces* and *Cladosporium* on the soybean biomass under salt stress.

Treatment	Shoot biomass	Root biomass
Control	30.10 ± 2.5	10.23 ± 1.51
*Talaromyces*	36.7 ± 2.18	12.25 ± 0.4
*Cladosporium*	38.2 ± 1.79	13.25 ± 1.02
*p* value	0.0025^**^	0.021^*^

^*^p < 0.05; ^**^p < 0.01; ^***^p < 0.001.

## Discussion

This study was conducted to reveal how salt stress affects the structure of the rhizospheric fungal community of salt-tolerant (Salt-R) and susceptible (Salt-S) soybean genotypes. In comparison to the Salt-S genotype, the fungal communities of the Salt-R genotype were higher in abundance and significantly different in structure, but not in diversity (Figures 1–3). The amount and type of root exudates secreted by different soybean genotypes was variable, which led to a diverse response to salt stress (Li et al., 2021). Therefore, Salt-R genotypes, except for secreting abundant organic acids directly to dilute NaCl, might also enrich some fungi with ability to secrete organic acids around the rhizosphere, thus increasing their resistance to salinity (Lian et al., 2020). Notably, salt stress usually amplified the segregating trend in fungal community structure between resistant and susceptible genotypes, which was in accordance with the observations of Lian et al. (2020) and Lian et al. (2020).

The composition of the rhizospheric fungal community was greatly altered by salt stress in both genotypes (Figure 3A). This was in line with previous studies that fungal community structure was influenced by the complicated effects of saline alkaline soil environments (Yao et al., 2021). There was also, however, genotype-dependance in the fungal community structure with non-salt stress (Figure 3A), which was in contrast with previous study (Wang et al., 2008). Wang et al. (2008) reported that fungal communities were not found to be significantly different among the three genotypes at the same growth stage (Wang et al., 2008). This phenomenon might be explained by the lower methodological resolution to test fungal communities (Gomes et al., 2003). It was possible that differences in soil physicochemical properties directly contributed to changes in the structure of fungal communities under salt stress (Figure 5). Moreover, Na^+^, Olsen-P, NH_4_^+^, NO_3_^−^ and pH were the most important factors that shaping the rhizosphere microbial community.

A number of studies have demonstrated that salt resistant genotypes had the ability of secreting some special root exudates to make plants more adapted to salt stress (Innes et al., 2004; Lian et al., 2020). We have previously shown that the resistant genotype can recruit beneficial bacteria and hypothesize that the same is true for fungi (Lian et al., 2019a). The relative abundance of several fungal taxa in the rhizosphere of Salt-R was higher compared to Salt-S under salt stress like *Talaromyces*, *Saitozyma* and *Cladosporium*. Moreover, *Talaromyces* and *Cladosporium* isolated from the rhizosphere soil were verified that significantly increased the shoot and root biomass of soybean (Table 4). It has been previously revealed that *Talaromyces* was able to solubilize phosphate at salinity and thus showed high tolerance to salt stress (López et al., 2020). Thus, *Talaromyces* may be a key species for improving salt tolerance in soybean. However, the other five genera have not yet been found to be associated with soil salt stress and their role needs to be investigated in more detail.

The two genotypes also showed differences in the relative abundance of specific trophic groups (Table 1). Saprotrophs were the dominant trophic mode in the present study. Saprotrophs, as the dominant guild, had the highest relative abundance in the Salt-R genotype under salt stress. It has reported that fungi belonging to saprotrophs might have an essential function in promoting nutrient conversion and controlling plant pathogens (Lian et al., 2019b). The increased abundance of saprotrophs is again directly linked to the presence of different *Talaromyces* species. Previous studies have well established that *Talaromyces* could facilitate plant growth through better utilization of nutrients by plants (Shi et al., 2022).

Co-occurrence network analysis revealed that network properties were inherently different among salt-resistant and susceptible genotypes (Figure 6; Table 2). Compared to Salt-R genotypes, there was fewer negative correlations and higher modularity in fungal networks of Salt-S genotypes under salt condition, according to network theory, probably because of weaker competitive relationships between microbial species within the rhizosphere (Saavedra et al., 2011; Fan et al., 2018). Additionally, Salt-R genotype exhibited a higher number of positive correlations than Salt-S genotype under salt stress, suggesting that most fungal members were connected through a series of cooperative relationships (Coyte et al., 2015; de Vries et al., 2018). However, this network structure was considered unstable because fungal members might be strongly influenced by environmental fluctuations, thus increasing unstable coupling (Coyte et al., 2015; de Vries et al., 2018). In addition, core species served as a critical pointcut to analyze how to alleviate salt stress (Table 3). For example, ASV23, ASV8, and ASV14 were identified as *Aspergillus*, which alleviated salt stress by producing organic acids to form organic acid-salt complexes (Ali et al., 2021). However, rhizosphere microbes also include bacteria, which can help soybeans resist salt stress by releasing hormones and promoting plant nutrient uptake, among other things, which cannot be ignored (Li et al., 2021). Bacteria should be explored in future studies and analyses in conjunction with fungi to explore the synergistic role of different microbial communities in helping the host to resist stress.

In conclusion, the rhizospheric fungal community of the two genotypes differed under salt stress. The Salt-R genotype recruited salt-resistant fungal species to the root zone to help alleviating salt stress. Different co-occurrence structure of the fungal community associated with the resistant genotype indicate more complex along with environmental changes. Taken together, the study provides new evidence for the important role of the soybean rhizosphere microbiome in conveying resistance to salt stress. In the future, the rhizospheric fungal community could serve as a promising breeding strategy to select for plants that are more resistant towards different stresses.

## Data availability statement

The datasets presented in this study can be found in online repositories. The names of the repository/repositories and accession number(s) can be found in the article/Supplementary material.

## Author contributions

DWH, MY, and LW: conceptualization, investigation, writing – review and editing, visualization, and supervision. MY, DZ, ZW, and ZZ: methodology. MY, HS, WW, and DZH: software. ZQ and BM: validation. JW: formal analysis. MY and DZ: data curation. MY: writing – original draft preparation, project administration, and funding acquisition. All authors contributed to the article and approved the submitted version.

## Funding

This work was supported by Scientific research business cost project of Heilongjiang provincial scientific research institutes (CZKYF2022-1-B020); China Agriculture Research System of MOF and MARA (CARS-04).

## Conflict of interest

The authors declare that the research was conducted in the absence of any commercial or financial relationships that could be construed as a potential conflict of interest.

## Publisher’s note

All claims expressed in this article are solely those of the authors and do not necessarily represent those of their affiliated organizations, or those of the publisher, the editors and the reviewers. Any product that may be evaluated in this article, or claim that may be made by its manufacturer, is not guaranteed or endorsed by the publisher.

## References

[ref1] Ali R. Gul H. Hamayun M. Rauf M. Iqbal A. Shah M.. (2021). Aspergillus awamori ameliorates the physicochemical characteristics and mineral profile of mung bean under salt stress. Chem Biol Technol Ag. 8:9. doi: 10.1186/s40538-021-00208-9

[ref2] Anderson M. J. (2001). A new method for non-parametric multivariate analysis of variance. Austral Ecol. 26, 32–46. doi: 10.1111/j.1442-9993.2001.01070.pp.x

[ref3] Bhatt K. Suyal D. C. Kumar S. Singh K. Goswami P. (2022). New insights into engineered plant-microbe interactions for pesticide removal. Chemosphere 309:136635. doi: 10.1016/j.chemosphere.2022.136635, PMID: 36183882

[ref4] Bulgarelli D. Schlaeppi K. Spaepen S. van Themaat E. V. L. Schulze-Lefert P. (2013). Structure and functions of the bacterial microbiota of plants. Annu. Rev. Plant Biol. 64, 807–838. doi: 10.1146/annurev-arplant-050312-120106, PMID: 23373698

[ref5] Coyte K. Z. Schluter J. Foster K. R. (2015). The ecology of the microbiome: networks, competition, and stability. Science 350, 663–666. doi: 10.1126/science.aad2602 26542567

[ref6] de Vries F. T. Griffiths R. I. Bailey M. Craig H. Girlanda M. Gweon H. S.. (2018). Soil bacterial networks are less stable under drought than fungal networks. Nat. Commun. 9:3033. doi: 10.1038/s41467-018-05516-7 30072764 PMC6072794

[ref500] Edgar R. C. Haas B. J. Clemente J. C. Quince C. Knight R. (2011). UCHIME improves sensitivity and speed of chimera detection. Bioinformatics. 27, 2194–2200. 21700674 10.1093/bioinformatics/btr381 PMC3150044

[ref7] Etesami H. Glick B. R. (2020). Halotolerant plant growth–promoting bacteria: prospects for alleviating salinity stress in plants. Environ. Exp. Bot. 178:104124. doi: 10.1016/j.envexpbot.2020.104124

[ref8] Fan K. Weisenhorn P. Gilbert J. A. Shi Y. Bai Y. Chu H. (2018). Soil pH correlates with the co-occurrence and assemblage process of diazotrophic communities in rhizosphere and bulk soils of wheat fields. Soil Biol. Biochem. 121, 185–192. doi: 10.1016/j.soilbio.2018.03.017

[ref9] Gomes N. C. M. Fagbola O. Costa R. Rumjanek N. G. Buchner A. Mendona-Hagler L.. (2003). Dynamics of fungal communities in bulk and maize rhizosphere soil in the tropics. Appl. Environ. Microb. 69, 3758–3766. doi: 10.1128/AEM.69.7.3758-3766.2003, PMID: 12839741 PMC165189

[ref10] Gu Z. Gu L. Eils R. Schlesner M. Brors B. (2014). Circlize implements and enhances circular visualization in R. Bioinformatics 30, 2811–2812. doi: 10.1093/bioinformatics/btu393, PMID: 24930139

[ref11] Hanin M. Ebel C. Ngom M. Laplaze L. Masmoudi K. (2016). New insights on plant salt tolerance mechanisms and their potential use for breeding. Front. Plant Sci. 7:1787. doi: 10.3389/fpls.2016.01787, PMID: 27965692 PMC5126725

[ref12] Hu L. Robert C. A. M. Cadot S. Zhang X. Ye M. Li B.. (2018). Root exudate metabolites drive plant-soil feedbacks on growth and defense by shaping the rhizosphere microbiota. Nat. Commun. 9:2738. doi: 10.1038/s41467-018-05122-7 30013066 PMC6048113

[ref13] Innes L. Hobbs P. J. Bardgett R. D. (2004). The impacts of individual plant species on rhizosphere microbial communities in soils of different fertility. Biol. Fert. Soils. 40, 7–13. doi: 10.1007/s00374-004-0748-0

[ref14] Jobst J. King K. Hemleben V. (1998). Molecular evolution of the internal transcribed spacers (ITS1 and ITS2) and phylogenetic relationships among species of the family Cucurbitaceae. Mol. Phylogenet. Evol. 9, 204–219. doi: 10.1006/mpev.1997.0465, PMID: 9562980

[ref15] Jones D. L. Willett V. B. (2006). Experimental evaluation of methods to quantify dissolved organic nitrogen (DON) and dissolved organic carbon (DOC) in soil. Soil Biol. Biochem. 38, 991–999. doi: 10.1016/j.soilbio.2005.08.012

[ref16] Kawai F. Zhang D. Sugimoto M. (2000). Isolation and characterization of acid- and Al-tolerant microorganisms. FEMS Microbiol. Lett. 189, 143–147. doi: 10.1111/j.1574-6968.2000.tb09220.x, PMID: 10930728

[ref17] Khasanov S. Kulmatov R. Li F. van Amstel A. Bartholomeus H. Aslanov I.. (2023). Impact assessment of soil salinity on crop production in Uzbekistan and its global significance. Agric. Ecosyst. Environ. 342:108262. doi: 10.1016/j.agee.2022.108262

[ref18] Kiers E. T. Duhamel M. Beesetty Y. Mensah J. A. Franken O. Verbruggen E.. (2011). Reciprocal rewards stabilize cooperation in the mycorrhizal symbiosis. Science 333, 880–882. doi: 10.1126/science.1208473, PMID: 21836016

[ref19] Li W. Godzik A. (2006). Cd-hit: A fast program for clustering and comparing large sets of protein or nucleotide sequences. Bioinformatics 22, 1658–1659. doi: 10.1093/bioinformatics/btl158, PMID: 16731699

[ref20] Li H. La S. Zhang X. Gao L. Tian Y. (2021). Salt-induced recruitment of specific root-associated bacterial consortium capable of enhancing plant adaptability to salt stress. ISME J. 15, 2865–2882. doi: 10.1038/s41396-021-00974-2, PMID: 33875820 PMC8443564

[ref21] Li X. Rui J. Mao Y. Yannarell A. Mackie R. (2014a). Dynamics of the bacterial community structure in the rhizosphere of a maize cultivar. Soil Biol. Biochem. 68, 392–401. doi: 10.1016/j.soilbio.2013.10.017

[ref22] Li X. Rui J. Xiong J. Li J. He Z. Zhou J.. (2014b). Functional potential of soil microbial communities in the maize rhizosphere. PLoS One 9:e112609. doi: 10.1371/journal.pone.0112609 25383887 PMC4226563

[ref23] Lian T. Huang Y. Xie X. Huo X. Shahid M. Q. Tian L.. (2020). Rice SST variation shapes the rhizosphere bacterial community, conferring tolerance to salt stress through regulating soil metabolites. mSystems. 5, e720–e721. doi: 10.1128/mSystems.00721-20 PMC7687028 33234605

[ref24] Lian T. Ma Q. Shi Q. Cai Z. Hai N. (2019a). High aluminum stress drives different rhizosphere soil enzyme activities and bacterial community structure between aluminum-tolerant and aluminum-sensitive soybean genotypes. Plant Soil 440, 409–425. doi: 10.1007/s11104-019-04089-8

[ref25] Lian T. Mu Y. Jin J. Ma Q. Cheng Y. Cai Z.. (2019b). Impact of intercropping on the coupling between soil microbial community structure, activity, and nutrient-use efficiencies. PeerJ. 7:e6412. doi: 10.7717/peerj.6412 30775180 PMC6369829

[ref26] López J. E. Gallego J. L. Vargas-Ruiz A. Peña-Mosquera A. L. Zapata-Zapata A. D. López-Sánchez I. J.. (2020). Aspergillus tubingensis and Talaromyces islandicus Solubilize Rock Phosphate Under Saline and Fungicide Stress and Improve *Zea mays* Growth and Phosphorus Nutrition. J. Soil Sci. Plant Nut. 20, 2490–2501. doi: 10.1007/s42729-020-00315-w

[ref27] Mendes R. Garbeva P. Raaijmakers J. M. (2013). The rhizosphere microbiome: Significance of plant beneficial, plant pathogenic, and human pathogenic microorganisms. FEMS Microbiol. Rev. 37, 634–663. doi: 10.1111/1574-6976.12028, PMID: 23790204

[ref28] Oksanen J. Blanchet F. G. Kindt R. Legendre P. Minchin P. R. O’Hara R. B., (2015). Vegan: Community Ecology Package. R Package version 22-21.

[ref29] Parente E. Cocolin L. De Filippis F. Zotta T. Ferrocino I. O'Sullivan O.. (2016). FoodMicrobionet: a database for the visualisation and exploration of food bacterial communities based on network analysis. Int. J. Food Microbiol. 219, 28–37. doi: 10.1016/j.ijfoodmicro.2015.12.001, PMID: 26704067

[ref30] Pathan M. S. Lee J. Shannon J. G. Nguyen H. T. (2007). “Recent advances in breeding for drought and salt stress tolerance in soybean” in Advances in Molecular Breeding Toward Drought and Salt Tolerant Crops. eds. Jenks M. A. Hasegawa P. M. Jain S. M. (Dordrecht, Netherlands: Springer), 739–773.

[ref31] Pauvert C. Buée M. Laval V. Edel-Hermann V. Fauchery L. Gautier A.. (2019). Bioinformatics matters: The accuracy of plant and soil fungal community data is highly dependent on the metabarcoding pipeline. Fungal Ecol. 41, 23–33. doi: 10.1016/j.funeco.2019.03.005

[ref32] Peltoniemi K. Straková P. Fritze H. Iráizoz P. A. Pennanen T. Laiho R. (2012). How water-level drawdown modifies litter-decomposing fungal and actinobacterial communities in boreal peatlands. Soil Biol. Biochem. 51, 20–34. doi: 10.1016/j.soilbio.2012.04.013

[ref33] Phang T.-H. Shao G. Lam H.-M. (2008). Salt tolerance in soybean. J. Integr. Plant Biol. 50, 1196–1212. doi: 10.1111/j.1744-7909.2008.00760.x, PMID: 19017107

[ref34] Qin Y. Druzhinina I. S. Pan X. Yuan Z. (2016). Microbially mediated plant salt tolerance and microbiome-based solutions for saline agriculture. Biotechnol. Adv. 34, 1245–1259. doi: 10.1016/j.biotechadv.2016.08.005, PMID: 27587331

[ref35] Saavedra S. Stouffer D. B. Uzzi B. Bascompte J. (2011). Strong contributors to network persistence are the most vulnerable to extinction. Nature 478, 233–235. doi: 10.1038/nature10433, PMID: 21918515

[ref36] Shi Q. Liu Y. Shi A. Cai Z. Lian T. (2020). Rhizosphere soil fungal communities of Aluminum-Tolerant and-Sensitive soybean genotypes respond differently to aluminum stress in an acid soil. Front. Microbiol. 11:1177. doi: 10.3389/fmicb.2020.01177 32547532 PMC7270577

[ref37] Shi X. Zhou Y. Guo P. Ren J. Zhang H. Dong Q.. (2022). Peanut/sorghum intercropping drives specific variation in peanut rhizosphere soil properties and microbiomes under salt stress. Land Degrad. Dev. 34, 736–750. doi: 10.1002/ldr.4490

[ref38] Tierney L. (2012). “The R statistical computing environment,” in Statistical Challenges in Modern Astronomy V. Lecture Notes in Statistics. eds. Feigelson E. Babu G. (New York, NY: Springer).

[ref39] Wang Q. Garrity G. M. Tiedje J. M. Cole J. R. (2007). Naïve Bayesian classifier for rapid assignment of rRNA sequences into the new bacterial taxonomy. Appl. Environ. Microb. 73, 5261–5267. doi: 10.1128/AEM.00062-07, PMID: 17586664 PMC1950982

[ref40] Wang G. Xu Y. Jin J. Liu J. Zhang Q. Liu X. (2008). Effect of soil type and soybean genotype on fungal community in soybean rhizosphere during reproductive growth stages. Plant Soil 317:135. doi: 10.1007/s11104-008-9794-y

[ref41] Wu Q. Sanford R. A. Löffler F. E. (2006). Uranium (VI) Reduction by *Anaeromyxobacter dehalogenans* Strain 2CP-C. Appl. Environ. Microb. 72, 3608–3614. doi: 10.1128/AEM.72.5.3608-3614.2006, PMID: 16672509 PMC1472366

[ref42] Yao R. Yang J. Zhu W. Li H. Yin C. Jing Y.. (2021). Impact of crop cultivation, nitrogen and fulvic acid on soil fungal community structure in salt-affected alluvial fluvo-aquic soil. Plant Soil 464, 539–558. doi: 10.1007/s11104-021-04979-w

[ref43] Yin J. Guo H. Ellen L. Jonathan R. Tang S. Yuan T.. (2022). Plant roots send metabolic signals to microbes in response to long-term overgrazing. Sci. Total Environ. 842:156241. doi: 10.1016/j.scitotenv.2022.156241, PMID: 35644397

