# Supplementary material for: Salt altered rhizosphere fungal community and induced soybean recruit specific species to ameliorate salt stress
Source: Front Microbiol. 2023 May 16;14:1142780. doi: 10.3389/fmicb.2023.1142780 (PMC10227517; doi:10.3389/fmicb.2023.1142780)
Supplement: Supplementary file 1 [file Table_1.DOCX]

**Salt-induced recruitment of specific fungal species to soybean to ameliorate salt stress**

Ming Yuan^1^, Di Zhang^1^, Zhen Wang^1^, Zhijia Zhu^1^, Haoyue Sun^1^, Wei Wang^2^, Dezhi Han^3^, Zhongcheng Qu^1^, Bo Ma^1^, Junqiang Wang^1^, Lianxia Wang^1^*, Dongwei Han^1^*

1.Qiqihar Branch of Heilongjiang Academy of Agricultural Sciences, Qiqihar, China

2.Institute of Soil Fertilizer and Environmental Resources, Heilongjiang Academy of Agricultural Sciences, Harbin, China

3.Heihe Branch of Heilongjiang Academy of Agricultural Sciences, Heihe, China

*Corresponding author1: Dongwei Han

Corresponding address: Qiqihar Branch of Heilongjiang Academy of Agricultural Sciences, Qiqihar, China

E-mail address: [handongwei126@126.com](mailto:handongwei126@126.com)

* Corresponding author2: Lianxia Wang

Corresponding address: Qiqihar Branch of Heilongjiang Academy of Agricultural Sciences, Qiqihar, China

E-mail address: wlx0427@163.com

**Supplementary Table S1** ASVs of Salt-R that are significantly abundance in the rhizosphere soil (0.3Salt-R VS 0Salt-R, *P* < 0.05)

| ASV | lock | pad | Phylum | Class | Order | Family | Genus | Species |
| --- | --- | --- | --- | --- | --- | --- | --- | --- |
| ASV1 | 6.54 | 3.51731E-06 | Basidiomycota | pad | Tremellas | Tremellas | *Sacituzumab* | Unclassified |
| ASV6 | 8.27 | 0.012134683 | Ascomycota | Sacituzumab | Axillaries | Axillaries | *Idriella* | Unclassified |
| ASV7 | 8.84 | 2.69415E-05 | Ascomycota | Eurotiomycetes | Eurotiales | Trichocomaceae | *Talaromyces* | *Talaromyces_helicus* |
| ASV9 | 2.63 | 6.3792E-05 | Basidiomycota | Tremellomycetes | Tremellales | Trimorphomycetaceae | *Saitozyma* | Unclassified |
| ASV17 | 2.31 | 1.22459E-05 | Basidiomycota | Wallemiomycetes | Wallemiales | Wallemiaceae | *Wallemia* | Wallemia_hederae |
| ASV19 | 5.70 | 4.33006E-07 | Ascomycota | Eurotiomycetes | Eurotiales | Aspergillaceae | *Aspergillus* | Unclassified |
| ASV21 | 3.63 | 1.05511E-06 | Basidiomycota | Wallemiomycetes | Wallemiales | Wallemiaceae | *Wallemia* | *Wallemia_hederae* |
| ASV24 | 6.05 | 0.000980071 | Basidiomycota | Tremellomycetes | Tremellales | Trimorphomycetaceae | *Saitozyma* | *Saitozyma_podzolica* |
| ASV29 | 7.07 | 8.67927E-07 | Ascomycota | Eurotiomycetes | Eurotiales | Trichocomaceae | *Talaromyces* | *Talaromyces_helicus* |
| ASV32 | 3.81 | 0.033391746 | Ascomycota | Saccharomycetes | Saccharomycetales | Saccharomycetales_fam_Incertae_sedis | *Candida* | *Candida_yuanshanica* |
| ASV38 | 1.96 | 0.008543475 | Basidiomycota | Tremellomycetes | Tremellales | Rhynchogastremataceae | *Papiliotrema* | *Papiliotrema_laurentii* |
| ASV80 | 2.74 | 0.003245407 | Ascomycota | Dothideomycetes | Capnodiales | Cladosporiaceae | *Cladosporium* | *Cladosporium_sphaerospermum* |

**Supplementary Table S2** ASVs of Salt-S that are significantly abundance in the rhizosphere soil (0.3Salt-S VS 0Salt-S, *P* < 0.05)

| ASV | logFC | padj | Phylum | Class | Order | Family | Genus | Species |
| --- | --- | --- | --- | --- | --- | --- | --- | --- |
| ASV7 | 9.37 | 1.39607E-05 | Ascomycota | Eurotiomycetes | Eurotiales | Trichocomaceae | *Talaromyces* | *Talaromyces_helicus* |
| ASV9 | 1.26 | 0.002042784 | Basidiomycota | Tremellomycetes | Tremellales | Trimorphomycetaceae | *Saitozyma* | Unclassified |
| ASV17 | 7.08 | 4.09869E-07 | Basidiomycota | Wallemiomycetes | Wallemiales | Wallemiaceae | *Wallemia* | *Wallemia_hederae* |
| ASV19 | 7.06 | 5.19084E-05 | Ascomycota | Eurotiomycetes | Eurotiales | Aspergillaceae | *Aspergillus* | Unclassified |
| ASV21 | 7.13 | 2.24138E-06 | Basidiomycota | Wallemiomycetes | Wallemiales | Wallemiaceae | *Wallemia* | *Wallemia_hederae* |
| ASV24 | 4.61 | 4.76891E-07 | Basidiomycota | Tremellomycetes | Tremellales | Trimorphomycetaceae | *Saitozyma* | *Saitozyma_podzolica* |
| ASV32 | 6.82 | 0.000955641 | Ascomycota | Saccharomycetes | Saccharomycetales | Saccharomycetales_fam_Incertae_sedis | *Candida* | *Candida_yuanshanica* |
| ASV38 | 3.98 | 0.000114374 | Basidiomycota | Tremellomycetes | Tremellales | Rhynchogastremataceae | *Papiliotrema* | *Papiliotrema_laurentii* |
| ASV44 | 4.21 | 0.000609314 | Basidiomycota | Tremellomycetes | Tremellales | Trimorphomycetaceae | *Saitozyma* | *Saitozyma_podzolica* |
| ASV63 | 6.12 | 6.462E-06 | Ascomycota | Dothideomycetes | Capnodiales | Cladosporiaceae | *Cladosporium* | Unclassified |
| ASV75 | 4.36 | 0.035386029 | Ascomycota | Saccharomycetes | Saccharomycetales | Saccharomycetales_fam_Incertae_sedis | *Candida* | *Candida_yuanshanica* |
| ASV96 | 3.16 | 0.00033123 | Ascomycota | Sordariomycetes | Hypocreales | Bionectriaceae | *Gliomastix* | *Gliomastix_roseogrisea* |

**Supplementary Table S3** The shared ASVs were found in both genotypes under salt stress

| ASV | Phylum | Class | Order | Family | Genus | Species |
| --- | --- | --- | --- | --- | --- | --- |
| ASV7 | Ascomycota | Eurotiomycetes | Eurotiales | Trichocomaceae | *Talaromyces* | *Talaromyces_helicus* |
| ASV9 | Basidiomycota | Tremellomycetes | Tremellales | Trimorphomycetaceae | *Saitozyma* | Unclassified |
| ASV17 | Basidiomycota | Wallemiomycetes | Wallemiales | Wallemiaceae | *Wallemia* | *Wallemia_hederae* |
| ASV19 | Ascomycota | Eurotiomycetes | Eurotiales | Aspergillaceae | *Aspergillus* | Unclassified |
| ASV21 | Basidiomycota | Wallemiomycetes | Wallemiales | Wallemiaceae | *Wallemia* | *Wallemia_hederae* |
| ASV24 | Basidiomycota | Tremellomycetes | Tremellales | Trimorphomycetaceae | *Saitozyma* | *Saitozyma_podzolica* |
| ASV32 | Ascomycota | Saccharomycetes | Saccharomycetales | Saccharomycetales_fam_Incertae_sedis | *Candida* | *Candida_yuanshanica* |
| ASV38 | Basidiomycota | Tremellomycetes | Tremellales | Rhynchogastremataceae | *Papiliotrema* | *Papiliotrema_laurentii* |
